# Increased Endocannabinoid Signaling Reduces Social Motivation in Intact Rats and Does Not Affect Animals Submitted to Early-Life Seizures

**DOI:** 10.3389/fnbeh.2020.560423

**Published:** 2020-12-09

**Authors:** Fernanda Teixeira Ribeiro, Marcia Ivany Silva de Serro-Azul, Fernanda Beraldo Lorena, Bruna Pascarelli Pedrico do Nascimento, Alexandre José Tavolari Arnold, Geraldo Henrique Lemos Barbosa, Miriam Oliveira Ribeiro, Roberta Monterazzo Cysneiros

**Affiliations:** ^1^Developmental Disabilities Postgraduate Program, Laboratory of Neurobiology and Metabolism, Mackenzie Presbyterian University, São Paulo, Brazil; ^2^Postgraduate Program in Translational Medicine, Federal University of São Paulo, São Paulo, Brazil

**Keywords:** sociability, seizures, endocannabinoid system, pilocarpine, autism (ASD), social reward processing, JZL195, CB1 receptor

## Abstract

The early life *status epilepticus* (SE) causes high anxiety and chronic socialization abnormalities, revealed by a low preference for social novelty and deficit in social discrimination. This study investigated the involvement of the endocannabinoid system on the sociability in this model, due to its role in social motivation regulation. Male Wistar rats at postnatal day 9 were subjected to pilocarpine-induced neonatal SE and controls received saline. From P60 the groups received vehicle or JZL195 2 h before each behavioral test to increase endocannabinoids availability. In the sociability test, animals subjected to neonatal SE exhibited impaired sociability, characterized by social discrimination deficit, which was unaffected by the JZL195 treatment. In contrast, JZL195-treated control rats showed low sociability and impaired social discrimination. The negative impact of JZL195 over the sociability in control rats and the lack of effect in animals subjected to neonatal SE was confirmed in the social memory paradigm. In this paradigm, as expected for vehicle-treated control rats, the investigation toward the same social stimulus decreased with the sequential exposition and increased toward a novel stimulus. In animals subjected to neonatal SE, regardless of the treatment, as well as in JZL195-treated control rats, the investigation toward the same social stimulus was significantly reduced with no improvement toward a novel stimulus. Concerning the locomotion, the JZL195 increased it only in control rats. After behavioral tests, brain tissues of untreated animals were used for CB1 receptor quantification by Elisa and for gene expression by RT-PCR: no difference between control and experimental animals was noticed. The results reinforce the evidence that the early *SE* causes chronic socialization abnormalities, revealed by the low social interest for novelty and impaired social discrimination. The dual FAAH/MAGL inhibitor (JZL195) administration before the social encounter impaired the social interaction in intact rats with no effect in animals subjected to early-life seizures.

## Introduction

The immature brain is highly susceptible to seizures given the fact that the excitation predominates over inhibition in the neuronal networks of the cortex and limbic structures, which is critical for brain development (Jensen and Baram, 2000; Rakhade and Jensen, 2009). Neonatal seizures, one of the most frequent neurological disorders in newborn infants (Saliba et al., 1996, 1999; Sheth et al., 1999; Mosley, 2010) may produce, *per se*, adverse developmental outcomes (Miller et al., 2002; Glass et al., 2009). As the evaluation of the long-term effect of early life seizures in clinical studies is difficult due to the number of variables involved in the outcome, the experimental model in rodents has been used for this purpose. Indeed, rodents of both genders subjected to early-life seizures exhibit a high-functioning autism-like phenotype, as chronic sociability abnormalities revealed by a deficit in social play behavior, low social preference for novelty and discrimination, elevated emotionality/anxiety-related behavior, impaired ultrasonic vocalization, and mild or no cognitive deficit depending on the task demand (Castelhano et al., 2010, 2013, 2015; Lugo et al., 2014; Bernard and Benke, 2015; Leite et al., 2016; Reynolds et al., 2016, 2017; Barbosa et al., 2017; Hodges et al., 2019; Mikulecká et al., 2019; Pacífico et al., 2020). Despite the well documented behavioral dysfunction, the mechanism underlying the impaired sociability remains unknown. Recently, we demonstrated that pilocarpine-induced neonatal *status epilepticus* (SE) leads to social motivation impairment, reduction in oxytocin receptor (OTR) expression in hippocampus concomitantly with an enhancement of oxytocin in hypothalamus (Pacífico et al., 2020). Mouchati et al. (2019) reported increased hyperconnectivity within the hippocampus (CA3–CA1) and between the hippocampus and PFC during the awake and sleep states as involved in impaired sociability. The hypothalamus and hippocampus are components of the social decision-making network (SDN), formed by the integration of the mesolimbic reward and social behavior networks (O’Connell and Hofmann, 2011). The hippocampus plays an essential role in social recognition through the OTR located in the anterior dentate gyrus and anterior CA2/CA3 regions of the hippocampal formation (Raam et al., 2017). Beyond the well-known role of oxytocin on social behavior, the endocannabinoid system has been gaining some attention.

The Endocannabinoid system is constituted by cannabinoid receptors CB1 and CB2, being CB1 the most abundant in the CNS, by endogenous ligands called anandamide (AEA) and 2-arachidonoylglycerol (2-AG) and by enzymes responsible for synthesis and inactivation (Mechoulam and Parker, 2013; Mechoulam et al., 2014). The brain distribution of CB1 receptors is consistent with its role on social-emotional functioning, being highly expressed in key parts of the SDN, including the central and basolateral amygdala, prefrontal cortex, hippocampus, dorsolateral striatum, ventral tegmental area, and the nucleus accumbens (Glass et al., 1997; Parsons and Hurd, 2015; Wei et al., 2017). Wei et al. (2017, 2015) reported that anandamide and oxytocin are intrinsically correlated in regulating social motivated behavior. Accordingly, oxytocin drives anandamide mobilization in the nucleus accumbens that regulates the socially motivated behavior through the activation of the CB1 receptor, whereas the pharmacological block of OTR stops this response. Moreover, the endocannabinoid system is also affected by epilepsy. Epilepsy leads to synaptic plasticity in the endocannabinoid system. For instance, Maglóczky et al. (2010) reported in adult mice subjected to pilocarpine-induced epilepsy as well in the human sclerotic hippocampus, but not in the non-sclerotic cases, an increase in the CB1 immunostaining in GABAergic axons of the dentate molecular layer, that was correlated to the pyramidal loss in the CA1 region of the hippocampal formation. Maglóczky et al. (2010) showed that the CB1 receptor plasticity is closely correlated to neuronal loss. However, differently than noticed in adults, chemoconvulsant-induced seizures during early development lead to minimal or no detectable neuronal loss, no exuberant synaptic reorganization and animals do not evolve to spontaneous seizure in adulthood (Stafstrom et al., 1992; Xiu-Yu et al., 2007). In this sense, no evidence is available whether the endocannabinoid system is affected by chemoconvulsant-induced early life seizures or if the endocannabinoid system is involved in impaired sociability in this seizure model. An understanding of this impairment could yield a potential target for the prevention and provide biomarkers for identifying or for monitoring this adverse outcome. Take as a whole, this study investigated the involvement of the endocannabinoids system on the impaired sociability following pilocarpine-induced early-life seizures.

## Materials and Methods

### Animals

All procedures were approved by the Mackenzie University Ethics Committee in Use of Animals (CEUA, 142/08/2016). The animals were maintained under controlled conditions (07:00–19:00 h, light/dark cycle; 23–25°C, a relative humidity of 50–55%). Newly born Wistar male rats were maintained with their mothers. Pups’ ages were determined from the day of birth (P0). Animals were weaned at P21 and housed in groups (3–4 animals per cage) with free access to water and food.

### Status Epilepticus Induction

SE is usually defined as continuous seizure activity lasting for 30 min or longer or intermittent seizures lasting 30 min or more from which the patient does not regain consciousness (Ilae Commission Report, 1997). Pilocarpine was used to induce SE as reported in previous studies of our group (Castelhano et al., 2010, 2013, 2015). The experimental group received pilocarpine (Sigma) 3.8% in saline (380 mg/kg, i.p.) on P9 (19–21 g) which corresponds to a full-term neonate, and the control group received saline solution (0.1 ml/10 g). The onset of SE occurred within 3–4 min following pilocarpine injection being characterized by head bobbing, intermittent myoclonic jerks, swimming, bilateral and asynchronous running movements of the legs, generalized tonic-clonic jerks, wild running, tonic extension of both the forelimbs with apnea (Haas et al., 1990). The mortality rate was up to 10%. Following cessation of SE (ca 3.5–4 h) animals returned to their mothers.

### Experimental Design and Drug Treatment

**Figure 1:**
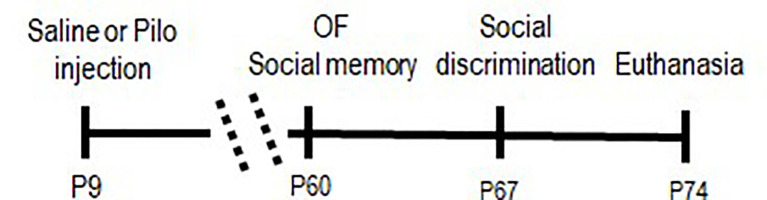
Timeline of procedures. Pilo, pilocarpine; and OF, open field.

At 21 days postnatal animals from four litters per main group, control and experimental, were randomly assigned to the following subgroups: CTRV, CTRJ, EXPV, EXPJ (Table 1). The vehicle was prepared by mixing 50 mM DMSO in 0.9% saline. From 60 days postnatal, young animals (230–250 g) received vehicle or the JZL195 (Tocris Bioscience^®^, dissolved in the vehicle, 0.01 mg/kg, i.p.) 2 h before each behavioral test. The treatment was administered twice for each animal, with 7 days interval between administrations to avoid any possible cumulative effect. The experimental design was defined as shown in Table 1 and Figure 1. JZL195 is a pharmacological inhibitor of enzymes FAAH and MAGL (these enzymes catalyze endocannabinoids, anandamide, and 2-AG). Administration of JZL195 increases AEA and 2-AG availability in the synaptic cleft. Additionally, 12 male rats (PN 30–35, 90–120 g) from three litters were used as social stimuli.

**Table 1:** Assignment of animals into groups and treatments.

Control group		Experimental group	
CTRV (11)	Animals received vehicle 2 h before the behavioral test	EXPV (11)	Animals received vehicle 2 h before the behavioral test
CTRJ (8)	Animals received JZL 2 h before the behavioral test	EXPJ (8)	Animals received JZL 2 h before the behavioral test

## Behavioral Procedures

The behavioral tests started from 60 days postnatal and were videotaped and scored by the researcher. Open Field and Social recognition tests were executed in sequence on the same day (P60–P64). Social discrimination test was executed one week after (P67–P70). The animals received JZL195 (0.01 mg/kg, i.p.) or vehicle 2 h before each test. The animals were transferred to the testing room 60 min before each daily session. The apparatus was cleaned with a 5% alcohol solution before each behavioral procedure. All behavioral tests were carried out in the same room with a controlled intensity of light (9 lx).

### Social Discrimination Test

The social behavior apparatus was adapted by Crawley (2007) and Novaes et al. (2012). Briefly, the sociability test, preceded by a habituation period, was divided into three sequential phases of 10 min each. During the habituation period, the test rat was placed in the middle chamber for 10 min with the retractable doors closed. Each of the two sides contained an identical empty wire cage. In the social approach phase, an unfamiliar rat was enclosed in one of the wire cages and the time spent in each compartment with objects or social stimulus was measured. In the social novelty phase, a new unfamiliar rat was enclosed into the wire cage in the opposite compartment and the time spent in each compartment was measured. It is important to mention that before the introduction of a social stimulus the test rat was trapped in the central chamber.

### Social Recognition Test

The social recognition apparatus was adapted by Guan and Dluzen (1994). For habituation, the animal test was placed for 3 min in the center of a white acrylic circular arena with 60 cm in diameter × 50 cm in height (Insight Limited, Brazil) concomitantly with an acrylic box (23.5 × 21 × 32 cm) containing small holes of 1 cm of diameter each in all four walls. After the habituation period, an unfamiliar conspecific with 30–35 days of age (social stimulus) was introduced into the acrylic box for 3 min. The procedure was repeated three times (trial 1 to trial 3) at intervals of 6 min. In the two subsequent sessions (trials 4 and 5), a new social stimulus of the same age as the previous one was used. It was measured the time of investigation toward a social stimulus. At intervals, the animal test was removed from the arena and the apparatus was cleaned with 5% ethanol.

### Open Field Test

The apparatus was a circular arena (60 cm in diameter × 50 cm in height) divided into 12 zones, being eight peripheral and four central (Insight Limited, Brazil). The animals were placed into the central area and observed for 10 min. The locomotor activity was expressed as the number of total lines crossed.

## Biomolecular Analysis

### Brain Tissue Preparation

At the end of the behavioral tests, untreated animals were decapitated under deep anesthesia (urethane 1.2 g/kg, i.p.), the brain was removed and the prefrontal cortex, amygdala, hippocampus, and striatum were dissected out and stored in a freezer −80°C until the time of analysis.

### Polymerase Chain Reaction (RT-PCR)

Frozen tissues were homogenized in Trizol reagent (Life Technologies) at a ratio of 1 ml Trizol/100 mg tissue. Briefly, total RNA was extracted from Trizol homogenates following the manufacturer’s instructions. The RNA was further purified using RNesay (Qiagen), quantified, and converted to double-stranded cDNA as per the manufacturer’s protocol.

The mRNA expression of the CNR1 receptor and house-keeping gene (B-actina) was performed using SYBR^®^ Green PCR Kit from Qiagen. The primers used for of CNR1 receptor and B-actina are as follows: Forward: 5′-CCATTTCAAGCAAGGAGCAC-3′, Reverse:5′ GTCATTCGAGCCCACGTAGA Foward: 5′-TTGCTGACAGGATGCAGA-3′, Reverse: 5′-ACCAATCCACACAGAGTACTT-3′, respectively (Jin et al., 2014). PCR amplification followed standard conditions: 5 min at 95°C, 40 cycles of 10 s at 95°C, and 30 s at 60°C. All reactions were performed in duplicate and the average of the duplicate was used. Relative gene expression was calculated using the 2^−ΔΔC_T_^ method according to Schmittgen and Livak (2008).

### Enzyme-Linked Immunosorbent Assay (ELISA)

CB1 quantification in the hippocampus, amygdala, prefrontal cortex, and striatum was assayed using a competitive enzyme immunoassay kit (ELISA) from Cloud-Clone. The procedures were performed following the protocol provided by the kit. Briefly, the brain tissue, free of blood, was sonicated with an ultrasonic cell disrupter till, homogenized in lysis buffer (w:*v* = 1:5), centrifuged for 5 min at 5,000× *g* and the supernatant assayed immediately. Samples, standards, and blank (100 μl) were loaded in duplicate into each well. The plate was sealed and incubated for 2 h at 37°C. After incubation, the liquid was removed and then 100 μl of Biotinylated CB1’s Detector Antibody was added. The plate was incubated for 1 h at 37°C. After incubation, the wells were washed three times, the Avidin-HRP Conjugate (100 μl) was added into each well and the plate was kept for 30 min at 37°C. After that, the wells were washed five times, the TMB substrate (90 μl) was added and the plate was kept at 37°C for 20 min. The reaction was stopped by the addition of 50 μl of stop solution. Immediately, the plate was reading on the spectrometer at 450 nm. The concentration of CB1 was determined from the standard curve, multiplied by the dilution factor, and normalized by total protein concentration.

## Data Analysis

Data were submitted to Kolmogorov–Smirnov and Shapiro tests of normality. Data that passed the normality test were analyzed using parametric tests. Sociability and social recognition test were analyzed by Mixed ANOVA using time of investigation (object × unfamiliar rat, familiar rat × social novelty) as within-subjects factor and groups (EXP vs. CTR) and treatment (vehicle vs. JZL195) as a between-subjects factor. Social recognition was also analyzed by Mixed Anova using sessions (1–3, 3–4, or 4–5) as within-subjects factor and groups (EXP vs. CTR) and treatment (vehicle vs. JZL195) as a between-subjects factor. Significant effects were probed with *post-hoc* testing (Bonferroni). The Open Field’s parameters were analyzed by two-way ANOVA, using Bonferroni for *post-hoc* testing. PCR-RT and Elisa were compared by Student’s *t*-test for independent samples. Data were expressed as mean + SD. *p*-values of 0.05 or less were considered significant.

## Results

### Sociability

**Figure 2:**
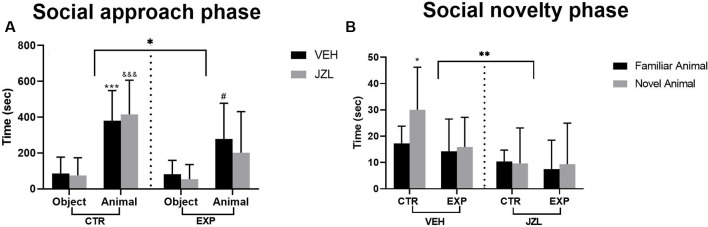
Time spent with the object or with the social stimulus **(A)** and with social stimuli (familiar × novelty) **(B)** are shown as mean ± SD. In **(A)**, animals spent more time with animals as compared to objects (***p = 0.0004, ^&&&^*p* = 0.0005, ^#^0.0226), but EXP group spent less time as compared to control (*p = 0.0168). In **(B)**, the JZL treatment decreased the time spent with social stimuli (**p = 0.0027). The untreated control group spent higher time with social novelty as compared with the familiar one (*p = 0.038). JZL impaired the control group’s social novelty preference (^#^*p* = 0.0113). EXP group, regardless of the treatment, exhibited a deficit in social discrimination.

In the approach phase, regardless the treatment, EXP animals spent less time than CTR exploring the compartments [(group effect, (*F*_(1,34)_ = 6.32, *p* = 0.0168)], but both groups animals spent more time in the compartment with unfamiliar rat as compared with object [stimuli effect (*F*_(1,34)_ = 45.31, *p* < 0.0001)]. No difference was noticed for any others parameters analyzed: [treatment effect (*F*_(1,34)_ = 0.354, *p* = 0.55), stimuli × group interaction (*F*_(1,34)_ = 3.95, =0.055), stimuli × treatment effect (*F*_(1,34)_ = 0.0007 *p* = 0.978), group × treatment interaction (*F*_(1,34)_ = 0, 92, *p* = 0.344), stimuli × group × treatment interaction (*F*_(1,34)_ = 0.425, *p* = 0.518]. The *post-hoc* test revealed significant difference between the time spent with object or with unfamiliar animal for CTRV (^&&&^*t* = 4.410, *p* = 0.0004), CTRJ (***t = 4.34, *p* = 0.0005) and EXPV (^#^*t* = 2.95, *p* = 0.0226; Figure 2A).

In the social novelty phase, a significant effect was noticed for treatment [vehicle × JZL, (*F*_(1,34)_ = 10.51, *p* = 0.0027)]. The treatment with JZL impaired the social discrimination specifically in intact animals. The *post-hoc* test revealed that the CTRV discriminated between familiar and novel animal (*t = 2.886, *p* = 0.038). EXP animals, regardless of the treatment, did not discriminate between the social stimuli. No difference was noticed for any other parameters analyzed [stimulus effect, (*F*_(1,34)_ = 2.59, *p* = 0.116), group effect, (*F*_(1,34)_ = 2.609, *p* = 0.1155), social stimulus × group interaction, (*F*_(1,34)_ = 0.776, *p* = 0.384), social stimulus × treatment interaction, (*F*_(1,34)_ = 1.897, *p* = 0.177), group × treatment interaction, (*F*_(1,34)_ = 1.245, *p* = 0.2723), social stimulus × group × treatment, (*F*_(1,34)_ = 2,037, *p* = 0.162; Figure 2A)].

### Social Recognition

**Figure 3:**
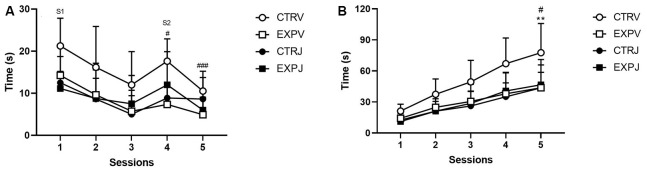
Time spent investigating social stimuli is shown as mean ± SD. In **(A)**, the time exploring the S1 decreased across sessions (1–3). Only the CTRV group showed enhancement in exploration time as S1 was replaced by S2 (^#^*p* = 0.0312) and habituation in the 5th session (^###^*p* = 0.0007). The JZL treatment decreased the exploration time of the control group, with no effect in the EXP group. In the cumulative frequency curve **(B)**, EXPV spent lower time investigating the social stimuli as compared to CTRV (**p = 0.0090), which time was unaffected by JZL treatment. The JZL significantly decreased the time of investigating of the control group (CTRV × CTRJ, ^#^*p* = 0.0189). S1 = social stimulus 1 and S2 = social stimulus 2.

In the social memory paradigm, the investigation toward the same social stimulus (S1) decreased across sessions 1–3 (*F*_(2,68)_ = 21.18, *p* < 0.0001). The *post-hoc* test showed that only the CTRV group, as the familiar animal was replaced by a novel stimulus (S2), increased the social investigation from trial 3–4 (^#^*t* = 2.828, *p* = 0.0312) and habituation in the 5th session (^###^*t* = 4.22, *p* = 0.0007). It was also noticed significant effect of the treatment (*F*_(1,34)_ = 5.703, *p* = 0.0226). The JZL195 decreased the investigation time of the control group, with no effect in the EXP group (Figure 3A). To analyze the motivation for the social encounter, the time of investigation was expressed as a cumulative frequency curve (Figure 2B). It was observed effect of group (*F*_(1,34)_ = 4.173, *p* = 0.0489), treatment (*F*_(1,34)_ = 4.228, *p* = 0.0475) and interaction between factors (group × treatment, *F*_(1,34)_ = 5.95, *p* = 0.0201). The effect of interaction suggests that JZL195 decreased the time of interaction specifically in the CTR group. The *post-hoc* test revealed that the overall interaction time was significantly lower in CTRJ as compared to CTRV (^#^*t* = 3.179, *p* = 0.0189), as well in EXPV as compared to CTRV (**t = 3.454, *p* = 0.009; Figure 3B).

### Open Field

**Figure 4:**
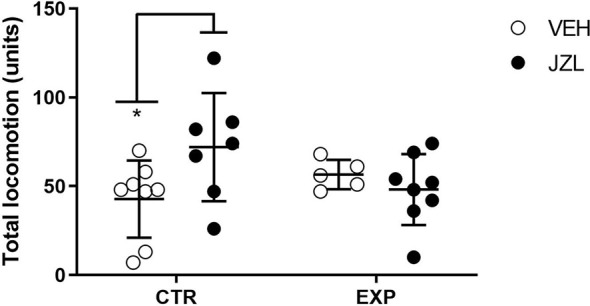
Total locomotion is shown as mean ± SD. JZL195 specifically increased the total locomotion of control animals (*p = 0.03).

To total locomotion, it was observed a significant effect of interaction between factors [treatment × groups, (*F*_(1,24)_ = 4.837, *p* = 0.037)], suggesting that one group was affected by the treatment. The *post-hoc* test revealed that JZL195 increased the locomotion in control animals (*t* = 2.537, *p* = 0.0359; Figure 4).

### Real-Time Polymerase Chain Reaction (RT-PCR)

**Figure 5:**
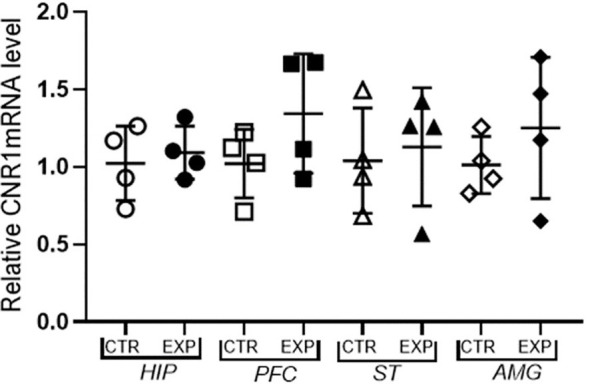
Expression of mRNA f CB1 receptor relative to the constitutive gene (Beta-actin) is shown as mean ± SD. No significant difference was noted between the control and experimental group.

The expression of mRNA of CB1 receptor in hippocampus (*t* = 0.467; *p* > 0.05), prefrontal cortex (*t* = 1.4; *p* > 0.05), striatum (*t* = 0.034; *p* > 0.05.) and amygdala (*t* = 0.97; *p* > 0.05) did not differ between control and experimental group (Figure 5).

### Enzyme-Linked Immunosorbent Assay (ELISA)

**Figure 6:**
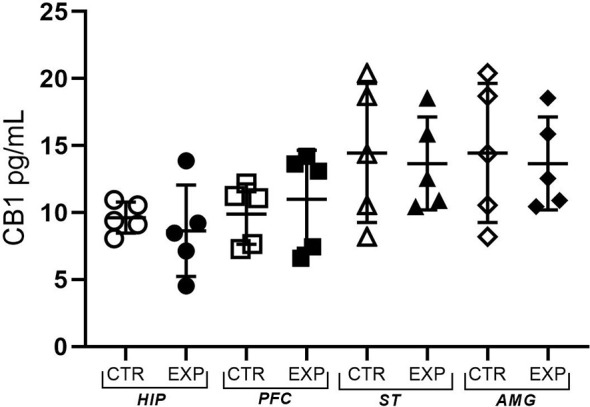
The expression of the CB1 receptor is shown as mean ± SD. No significant difference was noted between the control and experimental group.

CB1 receptor concentration in hippocampus (*t* = 0.65; *p* > 0.05), prefrontal cortex (*t* = 0.59 *p* > 0.05), striatum (*t* = 0.21; *p* > 0.05.) and amygdala (*t* = 1.37; *p* > 0.05) did not differ between control and experimental group (Figure 6).

## Discussion

This study investigated the involvement of the endocannabinoid system on the impaired sociability of young adult rats following pilocarpine-induced neonatal status epilepticus. For that, control and experimental groups were administered with JZL195, a FAAH and MAGL inhibitor that simultaneously increases AEA and 2-AG signaling inhibiting their hydrolysis. Wiskerke et al. (2012) showed that the JZL195 differentially enhances, in the nucleus accumbens, depolarization-induced elevation in dialysate levels of both AEA and 2-AG levels across time. The JZL195 effect was less pronounced on dialysate levels of 2-AG, and no difference was observed in the area under the curve (AUC) as compared to vehicle-treated controls. This effect is particularly relevant to the comprehension of the JZL195 behavioral effect in our study once the brain levels of AEA and 2-AG were not assessed.

As reported in previous studies, the animals subjected to pilocarpine-induced neonatal SE showed impaired sociability, reduced interest for social novelty, impaired discrimination, and reduced amount of time investigating the conspecifics, which was unaffected by the JZL195 treatment. Conversely, the JZL195 treatment decreased the sociability in intact animals, which under the JZL-195 effect showed impaired discrimination and reduced time investigating the conspecifics.

The complexity of the SDN must be taken into account for understanding the sociability impairment in this animal model, in the autism spectrum disorders either other psychiatric disorders and that alterations in these circuits or in the pathways that link these circuits may be underlying the deficits.

In this sense, a plausible interpretation for our findings is that in intact animals the JZL-195 preferentially increased the anandamide availability before the social encounter, leading to dampening the social motivation. Our assumption is based on the evidence of the AEA’s role in the social reward regulation in the striatum/nucleus accumbens. The social encounter with an unfamiliar conspecific increase the striatal AEA as compared to an encounter with a familiar conspecific or non-social controls (Marco et al., 2011). Wei et al. (2015) amplified this understanding, presenting new evidences that the oxytocin-dependent AEA signal contributes to the social reward regulation. The social encounter or the OT neurons activation in the paraventricular nucleus (PVN) of the hypothalamus drove the anandamide mobilization in the nucleus accumbens, being this effect blocked by OTR antagonists. Interestingly, the 2-AG levels were not affected by either intervention, reinforcing the oxytocin-dependent AEA role on social reward regulation. This important finding allows us to speculate that the lack of the JZL-195 effect in the experimental group may be related to disrupted OT signaling. Our group recently reported in this model a reduction in OTR expression in the hippocampus concomitantly with an enhancement of OT in the hypothalamus (Pacífico et al., 2020). Sun et al. (1996) also noticed an oxytocin and vasopressin elevation in rat hypothalamus following kainic acid-induced seizures. The OTR, abundantly expressed in the anterior dentate gyrus and anterior CA2 and CA3 pyramidal neurons of the hippocampal formation, are essential to social discrimination (Raam et al., 2017; Lin and Hsu, 2018; Tirko et al., 2018). Using an optogenetic approach, Raam et al. (2017) identified in the hippocampus an output from anterior CA2/CA3 regions to posterior CA1 as the pathway of social stimuli discrimination. These results are consistent with the recognized role of the hippocampus on social cognition regulation (Alexander et al., 2016; Okuyama et al., 2016; Danjo et al., 2018; Phillips et al., 2019; Tzakis and Holahan, 2019). This regulation is highly associated with the hippocampal posterior CA1 region projection to the prefrontal cortex, hypothalamus, and amygdala, key parts of the emotional reactivity and SDN (Hitti and Siegelbaum, 2014; Okuyama et al., 2016; Smith et al., 2016). Of note, the PFC constitutes the top-down control of social decision-making; the amygdala modulates the emotional processing; the hypothalamus, the stress modulation; the hippocampus, the memory processing, and the nucleus accumbens, the social motivation (Ko, 2017). Accordingly, we speculate that the decreased OT receptor expression in the hippocampus is underlying impaired social discrimination and that the disrupted hippocampal output to the hypothalamus impacted the oxytocin-dependent AEA release in the nucleus accumbens. Indeed, abnormal functional connectivity within the hippocampus (CA3–CA1) and between the hippocampus and PFC was reported in rats subjected to flurothyl-induced seizures from P5 to P14, which was associated with autistic-like behavior (Mouchati et al., 2019). Thus, it is not surprising that the integrity of the hippocampus is essential to social cognition, and that social impairment has been reported in patients (Wang et al., 2015; Ives-Deliperi and Jokeit, 2019), as well in animals with the temporal lobe of epilepsy (Marin et al., 2008) and animals with hippocampal lesions (Maaswinkel et al., 1996). Maaswinkel et al. (1996) reported that animals with lesioned hippocampus spent less time engaged in the social investigation than control rats and were unable to distinguish between a familiar and unfamiliar juvenile. Endocannabinoid system plasticity changes have been reported in the hippocampus in response to seizures (Wallace, 2004; Maglóczky et al., 2010), but no changes in the gene and protein expression of the CB1 receptor was noticed in our study. Perhaps, because that CB1 plasticity is related to the neuronal loss (Maglóczky et al., 2010) and no detectable neuronal loss is noticed following chemoconvulsant-induced early life seizures (Stafstrom et al., 1992; Xiu-Yu et al., 2007), suggests that the impaired sociability following neonatal SE is unrelated to CB1 receptor plasticity, rather than to the disrupted OT-dependent anandamide release.

Besides, we also noticed an increase in locomotor activity only in JZL-treated control rats. The effect of MAGL and dual FAAH/MAGL inhibition on motor behavior is still a matter of controversy. Seillier et al. (2014) showed that JZL195 in a dose-dependent manner decreased the total distance traveled in a new environment, as well as the selective MAGL inhibitor (JZL184). In JZL195-treated animals, the effect on motor behavior was inversely correlated with the 2-AG and AEA levels in nucleus accumbens, caudate-putamen, hippocampus, and prefrontal cortex. In contrast, in JZL184-treated animals, the locomotor activity was not correlated with 2-AG levels in any brain area, except for the caudate-putamen. The pretreatment with CB1 selective antagonist (SR141716A) prevented the JZL195 effect on locomotor activity, but it did not affect the JZL184, suggesting that JZL184 produces motor suppression through a CB1-independent mechanism. In contrast, Bedse et al. (2018) showed that JZL195 and JZL184 decreased both central zone activity and velocity, but increased the total distance traveled. Moreover, the JZL184, in a dose-dependent fashion, significantly increased brain 2-AG levels which was positively correlated with total distance traveled and negatively correlated with velocity. Similarly, JZL195 increased both brain AEA and 2-AG levels which were positively correlated with distance traveled and negatively correlated with velocity. The above studies showed the opposite effect on motor behavior despite they used similar doses of both inhibitors and the enhancement of brain endocannabinoids. At the present, we reinforce the argument of Bedse et al. (2018) when they mentioned that the effect of MAGL and dual FAAH/MAGL inhibition on locomotor activity is intriguing and needs further clarification.

The authors recognized the limitations of this study, that includes the lack of endocannabinoids assay in brain tissue, no utilization of CB1 agonist or antagonist, and no assessment of neuronal activity following the social behavior. Despite the limitations, the accumulated evidence so far provides insights into the hippocampus as a key element, which might not be limited to, in the social impairment following early-life status epilepticus.

## Conclusions

We present evidence that the early *status epilepticus* (SE) causes chronic socialization abnormalities, revealed by the low social interest for social novelty and impaired social discrimination. The dual FAAH/MAGL inhibitor (JZL-195) administration before the social encounter impaired the social interaction in intact animals with no effect in animals with chronic sociability abnormalities following early-life status epilepticus.

## Data Availability Statement

The raw data supporting the conclusions of this article will be made available by the authors, without undue reservation. The datasets generated for this study can be found in the following location: https://data.mendeley.com/datasets/nndnddwpjx/2.

## Ethics Statement

The animal study was reviewed and approved by Mackenzie University Ethics Committee in Use of Animals (CEUA, 142/08/2016).

## Author Contributions

All authors listed have made a substantial, direct and intellectual contribution to the work, and approved it for publication.

## Conflict of Interest

The authors declare that the research was conducted in the absence of any commercial or financial relationships that could be construed as a potential conflict of interest.
